# Design Innovation for Engaging and Accessible Digital Aphasia Therapies: Framework Analysis of the iReadMore App Co-Design Process

**DOI:** 10.2196/39855

**Published:** 2022-10-18

**Authors:** Tom Langford, Victoria Fleming, Emily Upton, Catherine Doogan, Alexander Leff, Daniela M Romano

**Affiliations:** 1 UCL Queen Square Institute of Neurology University College London London United Kingdom; 2 Department of Psychology and Language Sciences University College London London United Kingdom; 3 School of Information Studies University College London London United Kingdom; 4 Institute of Artificial Intelligence School of Computer Science and Informatics De Montfort University Leicester United Kingdom

**Keywords:** aphasia, reading impairment, co-design, framework analysis, speech and language therapy, digital health, accessibility

## Abstract

**Background:**

iReadMore is a digital therapy for people with acquired reading impairments (known as alexia) caused by brain injury or neurodegeneration. A phase II clinical trial demonstrated the efficacy of the digital therapy research prototype for improving reading speed and accuracy in people with poststroke aphasia (acquired language impairment) and alexia. However, it also highlighted the complexities and barriers to delivering self-managed therapies at home. Therefore, in order to translate the positive study results into real-world benefits, iReadMore required subsequent design innovation. Here, we present qualitative findings from the co-design process as well as the methodology.

**Objective:**

We aimed to present a methodology for inclusive co-design in the redesign of a digital therapy prototype, focusing on elements of accessibility and user engagement. We used framework analysis to explore the themes of the communications and interactions from the co-design process.

**Methods:**

This study included 2 stages. In the first stage, 5 in-person co-design sessions were held with participants living with poststroke aphasia (n=22) and their carers (n=3), and in the second stage, remote one-to-one beta-testing sessions were held with participants with aphasia (n=20) and their carers (n=5) to test and refine the final design. Data collection included video recordings of the co-design sessions in addition to participants’ written notes and drawings. Framework analysis was used to identify themes within the data relevant to the design of digital aphasia therapies in general.

**Results:**

From a qualitative framework analysis of the data generated in the co-design process, 7 key areas of consideration for digital aphasia therapies have been proposed and discussed in context. The themes generated were agency, intuitive design, motivation, personal trajectory, recognizable and relatable content, social and sharing, and widening participation. This study enabled the deployment of the iReadMore app in an accessible and engaging format.

**Conclusions:**

Co-design is a valuable strategy for innovating beyond traditional therapy designs to utilize what is achievable with technology-based therapies in user-centered design. The co-designed iReadMore app has been publicly released for use in the rehabilitation of acquired reading impairments. This paper details the co-design process for the iReadMore therapy app and provides a methodology for how inclusive co-design can be conducted with people with aphasia. The findings of the framework analysis offer insights into design considerations for digital therapies that are important to people living with aphasia.

## Introduction

### Background

Alexia is an acquired impairment of the ability to read, typically caused by a focal brain injury, such as that resulting from a stroke. People with alexia read slowly with substantial effort and make frequent word-based errors [1]. Some people experience alexia without other language impairments (pure alexia). More commonly, alexia occurs as part of a generalized language disorder known as aphasia, where the other domains of language (speaking, listening, and writing) may also be impaired. A third of stroke survivors develop some form of aphasia [2], and two-thirds of people with aphasia present with alexia [3]. The loss of reading ability can preclude many areas of life participation, such as socializing, working, and living independently. It is therefore not surprising that people with alexia report feelings of loss, frustration, and dissatisfaction [4,5].

Aphasia rehabilitation requires substantial hours (ranging from 20 to 100+ hours) of therapy to improve language abilities significantly [6-9]. Health care providers, however, are not always able to provide the level of specialized rehabilitation services required, and the National Health Service offers, on average, only 12 hours of aphasia therapy [10,11]. Perhaps unsurprisingly, almost half of stroke survivors report feeling abandoned following hospital discharge [12,13].

With an estimated 80 million stroke survivors globally as of 2016 [14] and an expected 25% increase in the number of stroke survivors by 2035 [15], there is a substantial need for health care providers to increase capacity for stroke rehabilitation services in order to meet the growing clinical demand. The adoption of digital technologies may offer a feasible solution to increasing individual therapy doses and may enable scalability to meet the increased service demands of larger stroke survivor populations in the years to come.

### iReadMore

**Figure 1:**
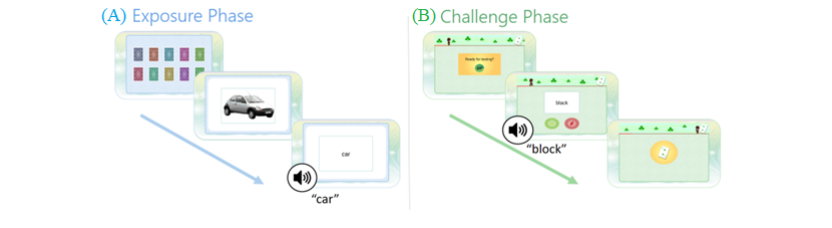
Therapy flow in the original iReadMore app design (prior to co-design). (A) Exposure phase that includes congruent pairings of written and spoken words on flashcards. (B) Challenge phase that includes both congruent and incongruent trials. In this example, there is an incongruent pairing of a written word and a spoken word, and the user would respond correctly by clicking the red “does not equal” button. The speaker icon denotes audio information.

iReadMore is a rehabilitation app that delivers single word reading therapy to train both reading accuracy and speed. It is intended to be used independently at home by people with alexia. The therapy involves mass practice of spoken-to-written word matching challenges with elements of gamification. The therapy has 2 phases (exposure phase and challenge phase). In the initial exposure phase, the user views 10 flashcards displaying congruent pairings of a written word, spoken word, and image. Following this, in the challenge phase, the user must decide whether a written word and a spoken word presented in unison are congruent or incongruent by clicking 1 of 2 buttons. The iReadMore therapy algorithm includes multiple parameters that personalize the difficulty level to suit the users’ reading abilities and keep the therapy challenging over time. This is achieved by altering the words that are presented in the therapy, the difficulty of each trial, and the amount of reading time provided for each trial. Figure 1 presents images of the therapy phases as seen in the trial version (prior to co-design). In this version, users did not receive information on their progress, such as reading test performance or therapy dose achieved.

A randomized controlled trial with 21 participants with poststroke alexia showed that iReadMore significantly improved word reading speed and accuracy following 4 weeks of therapy with an average dose of 34 hours, using the prototype app presented in Figure 1 [16]. Further research revealed that the therapy strengthened neural connectivity within the reading networks of stroke survivors [17].

Impairment-based interventions (such as iReadMore) can be effective and are well supported by a sound evidence base. However, the repetitive nature of these therapies can lead to some therapy users becoming disengaged or frustrated [18]. In the iReadMore trial, participants demonstrated significant clinical gains, and received support and motivation from the research team throughout the trial. However, informal feedback from participants highlighted the repetitive nature of the therapy and the low user acceptability of the app design. This put into question the ecological validity of the findings as a self-managed therapy. By employing a co-design approach to redesign the iReadMore app, we intend to innovate an effective therapy that is also accessible and engaging for users.

### Co-Design, Aphasia, and Digital Technology

Motivation is a key contributing factor in the success of stroke rehabilitation. For a self-managed therapy, maintaining long-term user motivation is vital to achieving the high therapy doses that are needed for therapeutic improvements [19,20]. A number of barriers to the uptake of digital therapies for people with aphasia are related to their communication impairment, co-morbidities, and level of prior experience with digital technologies [21,22]. One approach that can be used to improve the acceptability and accessibility of a therapy is to design it with the target user demographic, and this is known as co-design.

Co-design has been used in a number of digital applications for poststroke aphasia therapy in recent years [23-26]. EVA Park is an example of a co-designed therapist-led therapy delivered in an online virtual environment. It was found that users responded positively to the novelty of the co-designed therapy, as evaluated in terms of both a zero percent therapy dropout rate [24] and high acceptability deduced from qualitative interviews [27].

The perspectives of individuals with aphasia on literacy therapies have been explored in a handful of studies. Kjellén et al concluded that therapy design should be conducted in collaboration with people with aphasia, taking account of their personal goals and incorporating therapies into their daily life in a meaningful context [4]. The researchers also highlighted that people with aphasia felt “mechanical” therapies were not motivating enough, and the therapy content and mechanism need to be meaningful and interesting in order to stimulate recovery. Therefore, an effort is required to make aphasia therapies functional and personally relevant.

### Gamification

Gamification is an overarching term used to denote applying a diverse array of game design elements in nongame tasks in order to increase motivation and engagement. Increased levels of motivation can improve therapeutic outcomes for people with aphasia [19,20], and a number of studies have demonstrated positive clinical findings for aphasia therapies that were gamified [16,24,28-31]. Conroy et al reported anecdotally that users found their gamified therapy “especially engaging and motivating,” and the authors believed gamification contributed to the significant clinical gains by stimulating users’ executive and attentional functions, in addition to the speech production system, resulting in improved learning and retention [30].

More generally, a number of studies have found that commonly applied game design elements do not tend to appeal to older populations and can be regarded as either valueless or pressurizing [32,33]. However, the same game design elements will have different effects in different applications. Therefore, it is recommended to conduct context-specific research on gamification [34]. Despite the positive clinical findings mentioned previously, there is a lack of studies reporting on the views of people with aphasia regarding gamification in therapies. Thus, co-designing the gamification elements of therapy with the intended user group in the proposed research will provide further insights for developing self-managed therapies for people with aphasia.

### Objectives

We aimed to use a co-design approach to highlight a novel method for the inclusive redevelopment of an existing prototype therapy into a functional engaging therapy app that can be delivered at home and used independently by a person living with acquired alexia. In particular, we aimed to focus on key aspects of the user experience, including accessibility, gamification, and therapy engagement. By publishing this research, we hope to add to the growing literature on inclusive co-design and provide a case study for how co-design can be conducted in an inclusive manner.

By using a framework analysis of the data collected, themes were generated to better understand the requirements and desires of the user groups, which will be applied to inform the development of our future digital aphasia therapies.

## Methods

### Participants and Recruitment

Participants were recruited using stratified purposive sampling with convenience sampling through research group and institutional mailing lists, and other individuals known to the participants in this study. Participants included people with chronic alexia and their partners or carers. We aimed to get a diverse group of participants by stratifying for age, gender, experience with digital devices, and commonly co-occurring stroke morbidities, such as physical, visual, auditory, and cognitive impairments.

Table 1 reports the participant demographics. Twenty-five participants took part in 1 of 5 co-design sessions (4-6 participants per group). Participants varied in age from 29 to 78 years (mean 57, SD 12 years), and 52% (13/25) were female. Of the 25 participants, 19 had central alexia (alexia and aphasia), 3 had pure alexia and hemianopia, and 3 were partners or carers of someone with acquired alexia. With regard to prior experience with technology, 19 participants had a smartphone or tablet and 6 never owned a smartphone or tablet. Moreover, 10 participants had gained substantial experience using one of our digital therapies in a previous clinical trial.

**Table 1:** Demographic data of the participants who participated in the co-design group sessions.

Demographic		Value (N=25)
Female sex, n (%)		13 (52)
Age (years), mean (range)		57 (29-78)
**Diagnosis, n**		
	Central alexia (alexia and aphasia)	19
	Pure alexia	3
	No alexia (partner/carer)	3
**Prior technology experience, n**		
	Has a smartphone or tablet	19
	Has never owned a smartphone or tablet	6
	Previous participant in digital therapy app research	10

### Ethics Approval

Ethics approval for this study was granted by the University College London Research Ethics Committee (project ID: 15423/001). All participants provided written informed consent prior to commencement of the sessions.

### Study Design and Setting

Five in-person co-design sessions were held between June 2019 and January 2020 at the Institute of Cognitive Neuroscience, University College London in an accessible location. The sessions were facilitated by a multidisciplinary team of speech and language therapists (SLTs; VF and EU), a clinical psychologist (CD), and a medical design engineer (TL). All facilitators had completed professional training in qualitative health research at University College London or had prior experience in facilitating focus groups with people with aphasia. An app developer also observed the sessions. Sessions were limited to 4-6 participants to allow for group discussions without restricting each participant’s time to contribute. The number of sessions conducted was based on the iterative framework analysis process that was conducted after each session to reflect on whether subsequent sessions would be beneficial to further investigate the areas of interest. Sessions lasted between 1 and 2 hours, including breaks and time for refreshments. Further details are provided in Textbox 1.

Group discussions were held in a communal meeting room. When participants were testing the app prototypes, they could decide to do this in the meeting room using headphones or in a private side room, which provided less distractions. Semistructured questions were used to guide the discussions and were provided to all facilitators prior to the session. A framework analysis was conducted after each session to reflect on the discussions and develop the session guide and materials for the next session. Study reporting has been conducted in line with the COREQ checklist (Multimedia Appendix 2).

**Co-design focus group session structure.**

**Session structure**

The content of the sessions varied, but all contained the following core structure:

1. Welcome and introductions (5-10 minutes): Participants are welcomed and introduced to one another. Facilitators introduce themselves, and basic participation tips for the sessions are provided.
2. iReadMore instructions (5 minutes): Instructions for using the therapy are delivered by a member of the research team using a presentation and live demonstration, followed by answering questions from the group. In later sessions, this was replaced by an instruction video co-designed by participants, which was tested for inclusion in the app.
3. Independent use of the app (10-15 minutes): Following this, the latest prototype version of iReadMore therapy was tested on an Android tablet device, followed by an open discussion of the first impressions of the therapy.
4. Group discussion/ideation (20-40 minutes): Afterwards, discussions would lead into a problem and idea generation session, using a preplanned semistructured session guide.
5. Refreshments and open discussion (20-40 minutes): Finally, participants were offered refreshments and were able to talk freely. This gave participants the time to make any further points they would like and ask further questions in a less structured manner.

### Procedure and Co-Design

After participants were welcomed and provided informed consent, the aims of the co-design process were presented along with participation tips for the group discussion. Following this, participants tested the latest app prototypes by independently using the therapy with provided instructions. Facilitators would observe 1 or 2 participants’ interactions with the app. Facilitators assisted participants if required and made notes on any difficulties they were encountering.

Discussions began by asking participants about their experiences of testing the therapy prototype. This would then lead into a semiguided discussion based on preselected topics targeting key aspects of the therapy design, settings, functionality, interface, accessibility issues, and motivational/gamification concepts. Issues or difficulties raised during the interaction with the app acted as starting points for the co-design process, and participants then collaborated with each other and the facilitators to generate potential design solutions to address these issues. Where participants had a difference of opinion on the value of a design concept, an effort was made by the facilitators to see whether it could be refined in a way that led to a consensus. In addition, the mechanism of action of the therapy was not altered in the co-design process, as this was previously demonstrated to be clinically efficacious [16]. If a co-design concept could potentially preclude therapy effectiveness or participation for other users (eg, for those with visual or hearing impairments), it was highlighted and withdrawn from the process. The participants’ co-designed ideas were then developed further in collaboration with the research team and app developer using mock-ups and prototyping software, and taken to the following co-design session for the next group to try out.

In order to facilitate total communication and analysis of nonverbal output, the sessions were video recorded by 2 video cameras, and a variety of resources were available to participants, including paper, pens, visual analog mood scales, and printed visuals of the app. Questions to participants were also presented with visual aids to support comprehension. All notes and drawings made in the sessions were scanned and used alongside the video recordings and transcripts in the data analysis. To support the inclusion of participants with moderate to severe communication impairments, participants could bring a partner or carer, or be paired with a SLT to help facilitate participation. After the session, participants were contacted via phone or email to enquire if they had any further comments they wished to contribute.

Following the completion of the co-design group sessions, one-to-one beta-testing sessions were held to further refine the outcome of the co-design process and prepare the app for public release. This phase was conducted remotely due to the coronavirus pandemic. A further 25 participants were recruited through our mailing list and social media for the remote testing phase. Participants were provided with a tablet containing the iReadMore app or they downloaded iReadMore onto their personal device using the TestFlight app on iOS. Participants in this phase tested the app for a period ranging from 5 to 14 weeks and provided feedback on subsequent versions at monthly catch-ups and in between the assessments when issues arose.

### Data Collection and Analysis

Video recordings, notes, and drawings from participants and facilitators were analyzed using framework analysis, which utilizes a process of iterative refinement of themes in a data-driven approach [35]. Transcripts were developed from the session videos for annotation purposes. Both the videos and transcripts were analyzed to ensure nonverbal data (such as gestures and expressions) were not lost in the transcription process. Framework analysis was selected for its suitability in analyzing qualitative data at a group level in research that has a specific goal-based intention, such as co-design. There are 5 interconnected stages in framework analysis, and these were conducted in this study as described in Textbox 2. The analysis was conducted in Microsoft Excel (Microsoft Corp) by 2 researchers. Where disagreements occurred over codes, the 2 researchers discussed their conflicting interpretations and aimed to reach a consensus, potentially generating new codes as a result. Data saturation was discussed by the 2 researchers coding the data, who jointly decided when saturation had been achieved based on no further themes and codes being generated after the focus groups.

**Framework analysis methodology.**

**Framework analysis**

1. Familiarization: The data were studied in order to gain an insight into key concepts and recurrent themes. After each session, new data were analyzed. This allowed for initial codes and themes to be generated. After all sessions were complete, the data set was analyzed again in full.
2. Identifying a thematic framework: Emerging themes and subthemes were established and developed through discussions between the researchers. Data summaries were produced to represent the data in a succinct format.
3. Indexing: The generated codes and themes were applied to the data summaries. Although not part of the framework analysis, related quotes were also identified and sorted.
4. Charting: Data summaries were reorganized under the generated themes in the framework and rewritten in a more abstract manner to reflect the themes.
5. Mapping and interpretation: After charting, theme summaries were generated to represent the findings at a high level in the context of the research question. Descriptions and interpretations of the themes are presented below. Explanations and insights into the themes are considered in the Discussion section.

## Results

**Figure 2:**
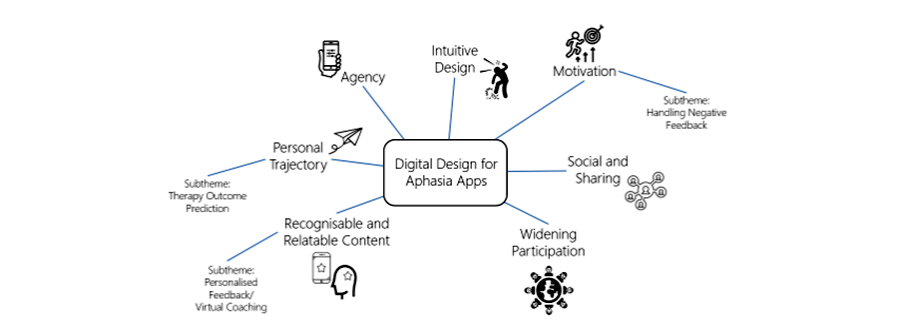
Thematic map displaying the themes and subthemes generated in the framework analysis of the iReadMore co-design process.

The framework analysis generated 7 distinct themes of key considerations for the design of a digital intervention for aphasia rehabilitation. The themes generated were agency, intuitive design, motivation, personal trajectory, recognizable and relatable content, social and sharing, and widening participation. Figure 2 displays a thematic map of the themes and key subthemes. The complete list of app features generated in the co-design process is listed in Multimedia Appendix 1.

### Theme 1: Agency

A prominent theme generated from the co-design process was to establish a stronger sense of agency for therapy users. Many participants mentioned the lack of control they felt in other aspects of their life as a result of their communication impairment and emphasized that restoring feelings of agency, even in small ways, was of significant value.

[On self-managed therapy] I think iReadMore is good because it gives X something for himself, something he can complete and be in control of, and I think that gives a big boost to his confidence.

Partner of a stroke survivor with aphasia, 70-year-old female

In practical terms, ways to increase agency that were suggested included giving users more control over therapy parameters and settings. Participants were interested in the workings of the therapy progression algorithm and suggested that an additional mechanism that allows users to adjust the therapy difficulty themselves would be valued as they could progress more easily to a difficulty level that suited them. Participants also preferred to decide their own therapy duration each day rather than have sessions of a fixed length. Further, it was mentioned that making the therapy easy to use without assistance would be empowering.

Notifications and pop-up reminders were viewed as superfluous and an annoyance, as users should know when to use the therapy and should know that performing the therapy is a significant activity in their daily lives, which should be motivated intrinsically by a desire to improve on their impairments. In specific circumstances, infrequent reminders would be more tolerable as long as they were providing useful information.

### Theme 2: Intuitive Design

Simplicity of the app design and ease of use were important considerations. Regardless of whether participants were experienced technology users, there was a unanimous preference for an app that was easy to pick up. Participants reported that difficulty in starting with a new therapy can lead to feelings of frustration and helplessness. In terms of iReadMore, the initial lack of clarity around where to tap on the screen during the exposure phase of the therapy led some participants to doubt their ability to use the therapy unassisted, while others felt frustrated. To resolve this, it was decided that a stronger visual contrast between clickable and nonclickable content would be needed, along with additional audio instructions and the use of animations to highlight fields that need to be clicked if no interaction is detected.

I think if you didn't get it immediately, because for me if I can't get something because of … things. I tend to give up and try something I can do. Because it'll make me feel better [laughs]

Stroke survivor with pure alexia, 46-year-old male

[On being unsure how to use an app] wouldn't have … confidence … to ask for help

Stroke survivor with aphasia, 65-year-old male

**Figure 3:**
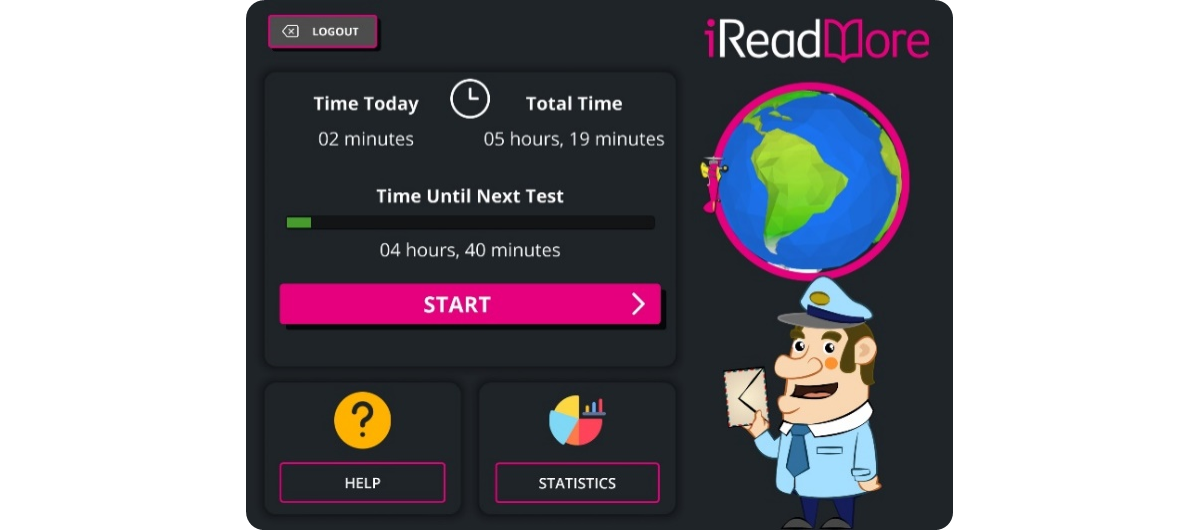
iReadMore main menu displaying therapy dose information; time to the next test; and buttons to start therapy, access help, and display statistics on therapy progress and test scores.

To further simplify the app experience, a more linear flow was implemented with buttons always present in the same locations. The visual appeal of the app design was of little or no importance to the majority of participants. Alternative designs for the main menu that involved more immersive and visually stimulating experiences were viewed as visually cluttered or difficult to interpret, with concerns about learning to use a more complicated app independently. Instead, a simplified more functional navigation to the therapy, help section, and feedback graphs was largely preferred (Figure 3).

### Theme 3: Motivation

Motivation unpinned many of the discussions in the co-design process. Participants thought that users of digital aphasia therapies do not need a lot of “bells and whistles,” as they are highly (intrinsically) motivated by the desire to improve on their impairments and do not respond enthusiastically to many traditional features of gamification aimed at improving extrinsic motivation.

*Colors make a big difference. For using everyday, I need something a bit fun* ***.*** *If it's a bit simple [gestures down with hands], but colors make it [gestures upward motion with hands]*

Stroke survivor with aphasia, 29-year-old female

One facilitator asked the following question:

Would it be demotivating to get negative feedback?

The response was as follows:

No, no. For me personally, if I’m getting it wrong but going forward, then I'm going forward … good for my understanding.

Stroke survivor with aphasia, 56-year-old female

Some did not understand the gamification concepts (such as points, high scores, avatars, and badges) or their intended purposes, while others felt they were not of value for this demographic.

**Figure 4:**
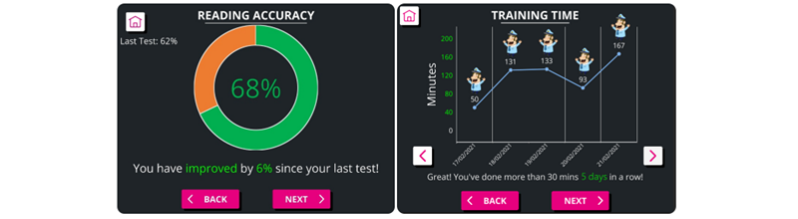
iReadMore feedback graphs and personalized messages for (A) reading test accuracy and (B) training time. On the graph, the stickers denote each day where 30 minutes of therapy were completed.

Participants thought that features to support motivation were needed later in the therapy to maintain usage over weeks to months. They proposed that the main driver of motivation long term was the ability to track and interpret their own therapy progress using the in-app reading tests, which are completed after every 5 hours of therapy. Many styles of presentation for this information were discussed and prototyped. The final designs were highly visual, with minimal lexical information and multiple representations of the scores to increase accessibility (Figure 4).

**Figure 5:**
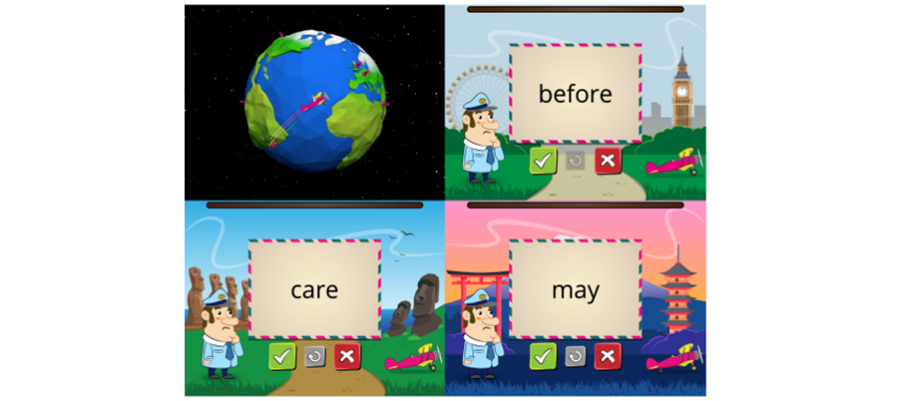
Therapy design travel concept.

Adding in visual novelty was seen as another way to maintain interest and denote progression through the therapy. Therefore, a number of designs were suggested, and finally, a travel-based concept with 10 destinations that users fly to around a 3-dimensional world was implemented (Figure 5). As such, when users complete 20 minutes of therapy, they visit a new destination. Users were advised to use the therapy for 30 minutes a day, so that they would visit a new location at least once a day at this rate. The destination backgrounds in the therapy were static to prevent distraction from the therapy task, and they acted as borders without text elements or animations.

The concept of receiving negative feedback was a key subtheme in the discussions of the workshops, with varied responses from participants. When asked about how they responded to the negative feedback, many believed it was acceptable and appropriate. Some thought it was key to motivating them to improve and was part of the process. However, 1 participant reported that he would like the option to hide the test results depending on his mood. The participant felt that being confronted by the impairment too often would be demotivating or upsetting, making him less likely to engage with the therapy. All agreed that being able to choose was a beneficial addition to the therapy, and as such, test results could be viewed by clicking on the “Statistics” button on the main menu (Figure 3).

### Theme 4: Personal Trajectory

Clear and consistent perspectives from participants were that stroke survivors with alexia are on individual journeys of rehabilitation and that gamification concepts of competition, leaderboards, and other comparisons between users are viewed negatively and are seen as detrimental to user motivation. Collaboration was also seen as pressurizing due to negative feelings arising from letting others down. Instead, participants wanted to focus on their personal progress in the therapy through regular feedback and praise for consistent use of the therapy.

Everyone has a different rate of improvement … So therefore, you don't want to benchmark yourself against others… I think the challenge is with you and progressing where you are and what you can do.

Stroke survivor with aphasia, 75-year-old male

A subtheme of whether being able to predict an individual’s future therapeutic outcomes was of value had a mixed response from the groups. There were concerns over inaccuracies as well as denial of service if it appeared it would not be beneficial. Participants reported they would prefer to try the therapy and decide whether it is not working for themselves or decide collaboratively with their clinician. However, it was also suggested that predictions could be a useful motivational tool to inspire users to continue progressing with the therapy if they were reported after the interval reading tests to motivate users to continue with the therapy. This concept will be explored further in future work looking into the feasibility of in-app therapy prediction.

### Theme 5: Recognizable and Relatable Content

This theme relates to participants’ preferences on how information is presented in the app. It was thought that a large proportion of digital therapies were designed with a young demographic in mind. However, a surprising outcome for the researchers was the pervasiveness and appeal of emoticons (emojis). Participants reported using emojis in place of words when they were having word-finding difficulties.

Because it feels quite young, it doesn't make you feel good about doing the exercise. It makes you feel like your level of understanding is lower

Stroke survivor with pure alexia, 46-year-old male

Yes, it suggests you’re doing this at school and not as an adult. It needs to be something that we're accustomed to seeing and understanding.

Stroke survivor with pure alexia, 78-year-old male

**Figure 6:**
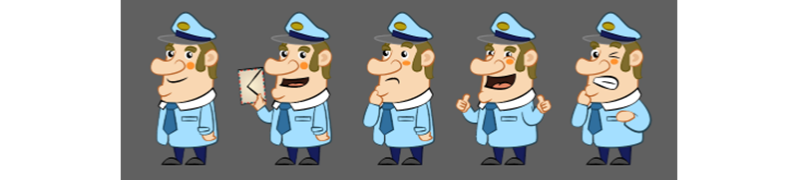
iReadMore character design and challenge phase feedback reactions.

Some participants did not understand or engage with the gamification concepts of points and scores. Further, some individuals had difficulty in number reading and found numerical scores distracting when incorporated into the therapy, so these were removed. The numeric point system was replaced with visual and audio content delivered through an animated cartoon character (Figure 6) to provide immediate performance feedback on a therapy challenge.

Participants thought the language used in instructions in the app and guidance for using the therapy should be simple and unambiguous. A couple of participants referred to frustration from not receiving clear guidance on how to use a therapy effectively. The group felt that quantified realistic goals would inspire regular use and confidence that they are using the app correctly. Ambiguous guidance, such as “use the therapy as much as you can,” was seen as unhelpful. One participant described that previous experiences of using therapies for long continuous periods in the first instances led to fatigue and would not be feasible longer term. On the other hand, clearer guidance, such as “use the app for 30 minutes a day,” was seen as motivating, achievable, and providing evidence-based advice. Therefore, this was implemented in the app.

Exploration of implementing a virtual coach in the app received mixed feedback. Some participants thought this would distract from the therapy or overcomplicate what users would like to receive from the therapy. However, the implementation of personalized positive feedback without the embodiment of a virtual coach was unanimously supported. Examples of feedback included how often participants were using the app, their performance, and their overall progression in the therapy in terms of reading accuracy and speed test scores. Participants felt that once or twice a week was an appropriate frequency for these types of messages and that it needed to feel sporadic and related to their personal performance.

### Theme 6: Social and Sharing

Participants wanted to be able to share their therapy progress with personal contacts and clinicians. Many participants were eager to incorporate a screenshot, which they could share with their family and friends to share their therapy progression. One participant mentioned that it could help to act as an icebreaker and enable open discussion about their condition, something which they currently find difficult to do. Only a few participants wanted to be able to share this feedback on social media. Many wanted to share this information with close personal contacts, either in person or via email, text message, or a messaging app, such as WhatsApp.

When I finish and go ‘yay!’, I want to show my family. [Picks up phone and opens WhatsApp] I love send photos!

Stroke survivor with aphasia, 60-year-old female

Would be great to show to my therapist. That way she’ll know that I’m actually doing the home practice! [laughs]

Stroke survivor with pure alexia, 50-year-old female

The other aspect of this theme was being able to share information with their clinicians, in particular, SLTs, or with facilitators and group members at their aphasia support groups. This was suggested as a feature that would be an additional benefit of using the app, as it could demonstrate their therapy compliance and progression, which could be used to report competence and willingness. Further, 2 participants mentioned that this could aid discussions with their clinical team over clinical decision-making, where the SLT could advise on whether the therapy is working for them.

### Theme 7: Widening Participation

The final theme relates to accessibility barriers for digital therapies. Issues relating to usability of the app in the context of aphasia as well as prevalent co-morbidities, such as physical (hemiplegia and hemiparesis), visual (hemianopia, color blindness, and visual neglect), auditory (high frequency hearing loss), and working memory impairments were raised. Based on these, the groups developed design refinements that would make the app more accessible. For example, the app does not require using more than one finger to operate and does not need to be held while in use, buttons and important visual content are always located centrally on the screen, the words in the therapy are read out twice in both female and male voices, and if no response is detected, spoken instructions are repeated and, in some cases, highlighted on the screen through animations.

Can’t do! When you first start, you need to focus on the word… and don’t want distractions. Not for me with distractions, not for me.

Stroke survivor with aphasia, 38-year-old female

An early prototype used animations throughout the therapy trials to make it more visually stimulating; however, this prevented a number of participants from knowing where to focus on the screen and was regarded as a distraction. As a result, animations were limited to reporting feedback after the user has answered a trial, as a balance between making the therapy visually stimulating and minimizing distractions.

Another significant barrier to access arose from minimal prior experience with technology. Issues were related to the technical difficulties of setting up and using a tablet device, and downloading the therapy. In response, aphasia-friendly instructions and frequently asked questions that were generated in the co-design process were integrated into the app. Participants wished to be able to contact the team directly for technical support or guidance. Therefore, an anonymous “Contact us” button was added to the “Help” section of the app. This allowed the research team to assist users whilst maintaining anonymity in line with our ethical approval and data security regulations.

Finally, concerns were raised over deploying the app solely on Android tablets as initially intended due to financial constraints. Some participants were unsure of what kind of device was required to use the therapy. The majority of the group did not have a tablet at home, and the minority that did were split between Apple devices and Android devices. As a result, the app was developed for Apple and Android phones and tablets.

## Discussion

### Overview

Conducting a framework analysis alongside co-design allowed for the dual development of app design and qualitative themes in a way that was synergistic and efficient.

The inclusive co-design methodology highlighted the need for a number of additional features in the app that had not been previously considered by the researchers. They arose from the designs and discussions of the participants, which were novel and informative. The iterative phases of co-design allowed us to not only capture the comments and reactions to a particular aspect of the app, but also verify that the redesign was congruent with the participants’ expectations. In this way, co-design can be a useful tool for stepping out of the traditional paper-based or clinician-led therapy tasks and innovating new therapies that go beyond what is achievable without technology.

### Themes

It was particularly pertinent for participants to promote a sense of agency in the therapy, which they may be lacking elsewhere. In the sessions, participants mentioned that not being able to use digital therapies, which are specifically aimed at their demographic, led to feelings of inadequacy and low competence, and prevented further engagement with those therapies. Recently, another study reported similar findings on the impact that digital technologies can have on feelings of agency and self-identity for people with aphasia [36]. On the other hand, digital therapies that can effectively be used independently were reported to have positive effects on personal empowerment and routine building.

The visual appeal of the app content was found to not be a primary concern for many participants. This finding is in contrast with previous findings on co-designed digital therapies, such as EVA Park [37] and GeST [25], both of which utilize immersive virtual worlds. We found that our participants preferred simpler navigation and intuitive app flow with less overtly gamified approaches to therapy. This could be due to fundamental differences in the therapy delivery, as EVA Park and GeST are SLT-led therapies for communication production. Co-design is by nature context-specific research, and therefore, it can be expected to produce contrasting findings for different applications. In our case, participants may have been prioritizing ease of use over immersion in the context of a self-managed therapy. However, visual (nonlexical) communication underpins many of the aspects on effectively communicating feedback through graphical or symbolic means.

Maintaining motivation was reported to be driven by intrinsic motivation and self-monitoring reading improvements through graphs or personalized messages. When participants were presented with variations of gamified therapy prototypes aimed at promoting extrinsic motivation, it was often felt that these alone would have little impact on their decision to use the therapy. The subtheme on receiving negative feedback was in contrast with the concept of errorless learning, which is often applied in rehabilitation technologies, and more in line with error-reducing learning [38]. However, it may be important to consider that people with aphasia who actively take part in research may display higher intrinsic motivation than those who do not. Many of these participants had taken part in previous studies involving highly gamified digital therapies, and this may have shaped their perspective. Therefore, the findings may not relate to the experience of people with aphasia and lower intrinsic motivation. In order to try and gain a wider perspective in future work, all users of the therapy will be able to anonymously provide qualitative feedback through the app.

Discussions on integrating recognizable and relatable content have similarities with design concepts being explored in other aphasia therapies, such as Web ORLA, which utilizes an embodied virtual therapist in the program [39]. Within the timeframe and financial limits available for this research, exploring the implementation of a virtual coach in iReadMore was deemed unfeasible, and personalized feedback on therapy usage and progress was seen as an appropriate alternative to this (Figure 4). There were also concerns it may lead to accessibility issues that could preclude some users from being able to engage with the therapy due to the technical and linguistic requirements of communicating with a virtual coach. Research exploring the feasibility of applying virtual coaches in rehabilitation for older adults, including people with aphasia, is ongoing [40]; however, this study also excluded those with global aphasia.

The emphasis on integrating social opportunities into the therapy is an understudied and somewhat underutilized concept in digital therapies at present, and participants generally felt this was a key area for improvement. This relates to previous research, which has found that people with aphasia tend to have a reduced social network and less frequent social interactions [41] while also experiencing an overall reduction in quality of life compared to stroke survivors without aphasia [42]. It was noted by the researchers that the participants who felt they would not want to see their own progress (as highlighted in the motivation theme) also did not want to share their progress with a clinician or friends and family. Their focus was on making the app independently and privately usable, whereas other participants wanted features that would enable real-world connections by sharing this information to prompt conversations about their condition with friends and family. Therefore, a balance is required to appeal to these conflicting perspectives. However, there are also a number of obstacles to integrating aspects of the social and sharing theme into a digital therapy, including concerns of data security, regulatory affairs, content moderation, and the complexity of the design required, which will need to be considered.

The theme of widening participation has parallels to the findings of a recent clinical review of technology use in aphasia [43]. This survey revealed that people with aphasia are more likely to have access to a tablet device than a mobile phone or computer. However, the population assessed was currently receiving speech and language therapy, and it was more likely that the tablet was owned by the clinical service than the person with aphasia. Therefore, in order to reach people who are not currently receiving speech and language therapy, it is important to release the application on tablet and mobile devices across platforms, and in the future, it is important to develop a desktop version of the app.

A number of themes generated in this study have theoretical underpinnings in the self-determination theory [44,45]. The themes of agency, motivation, social and sharing, and personal trajectory all relate to fulfilling aspects of the fundamental psychological needs of autonomy, competence, and relatedness as proposed by the theory. This theory is often applied to health intervention and gamification research and has significant parallels with theories of motivation specific to aphasia rehabilitation literature, such as person-centered life participation [46], and social approaches [47] to aphasia intervention, which both have parallels with the social and sharing theme in particular. 

### Reflections and Future Work

This study reinforces the current literature on the ability to successfully conduct a co-design study with people with aphasia. A core component of the co-design process is establishing total communication techniques that enable participants to engage meaningfully. These techniques include incorporating drawing, writing, gesturing, visual aids, and emotion scales in the co-design sessions [48]. It can be beneficial to know the communication profiles of participants ahead of time in order to support specific communication needs and explore how participants can be best supported to contribute [49]. In addition, involving carers and partners in the co-design sessions can further enable effective communication, particularly for individuals with more severe impairments [50]. Finally, the technique of asking participants to consider the perspectives of other individuals with aphasia who they knew personally was particularly useful in addressing issues, which would form the basis of the widening participation theme. Participants were asked to think of other individuals they knew with alexia or aphasia, and were asked what would help make the therapy accessible and appealing to them. Additionally, participants were asked to reflect on other apps that they use for therapy purposes or use generally.

The implications of the COVID-19 pandemic led us to conduct testing remotely in people with aphasia using the therapy at home, with their own devices where possible. Testing the therapy in the same setting as it is intended to be used was highly valuable and enabled the inclusion of participants outside of our usual catchment area as an added benefit. Stratifying users by technology usage and prior participation in a digital therapy clinical trial was important for ensuring the development of an app that was accessible to first-time users while also remaining engaging after use for a substantial period of time required to achieve therapeutic gains. However, we found similar trends for both those with and without prior technology experience in wanting to prioritize the ease of use of the app over design novelty or complexity. This was in order for users to feel confident in using the app independently, as the frustration of not knowing what to do with a digital therapy was highlighted as a key reason for therapy disengagement.

Design changes as suggested here have been implemented into the app, and the app has been released on the Apple App Store and Google Play Store. A mixed methods roll-out trial (NCT04849091) has been started to evaluate the clinical effectiveness of the app for real-world users, with study registration and data collection being conducted entirely through the app. Further research will involve a trial of iReadMore in people with a reading impairment resulting from primary progressive aphasia, a language-led dementia [51].

### Conclusions

This study offers tangible rationale to support the application of inclusive co-design procedures for persons with reading and language impairments, and elucidates the methods used. The findings of the framework analysis offer insights into design aspects that are important to people living with alexia and aphasia in the innovation of digital therapies. The co-designed version of the iReadMore app is available now for use in the rehabilitation of acquired reading impairments.

## Appendix

Multimedia Appendix 1 iReadMore design changes.

Multimedia Appendix 2 COREQ (Consolidated Criteria for Reporting Qualitative Research) checklist.

## Abbreviations

SLT: speech and language therapist
