# Supplementary material for: Design Innovation for Engaging and Accessible Digital Aphasia Therapies: Framework Analysis of the iReadMore App Co-Design Process
Source: JMIR Neurotechnol. 2022 Oct 18;1(1):e39855. doi: 10.2196/39855 (PMC12671282; doi:10.2196/39855)
Supplement: Multimedia Appendix 1 [file neuro_v1i1e39855_app1.pdf]

Design Innovation for Engaging and Accessible Digital Aphasia Therapies: A Framework Analysis of the iReadMore App Co-design Process

Appendix 1 – iReadMore Design Changes

iReadMore design changes organised by theme. A green symbol (●) indicates the design change has already been implemented in the app, orange (●) indicates we are currently exploring implementation subject to time and funding availability, and red (●) indicates this design change was deemed unfeasible or beyond the scope of this work.

Theme 1 – Agency

- Design app to be useable independently by persons with aphasia ●
- Add in feature to set therapy session duration using a sliding scale when pressing start therapy ●
- User-led therapy difficulty progression mechanism to increase the therapy difficulty if the user feels like progress is too slow ●
- Provide users with more fine-tuned settings to:
    - Turn off/on sound effects ●
    - Turn off/on background visuals ●
    - Turn off/on animations ●

Theme 2 – Intuitive Design

- Simplified main menu design ●
- Consistent button design throughout app and use of visual highlights and animations to denote where to tap or fields that need completing ●
- Replace email and password log-in with accessible one-click Google authentication system ●
- Automatically enable volume on when opening the application ●
- User can tap the card to reveal the word again in addition to pressing the Repeat button ●
- Location of action buttons will be consistent throughout the app navigation ●
- Add in pause therapy feature ●
- Simplification of exposure phase buttons to make knowing where to tap more intuitive ●
- Replacing the animated therapy bar character with a simple therapy progress bar for ease of understanding ●

Theme 3 - Motivation

- Implementation of three feedback graphs in a page accessible from the main menu ●
- Visual therapy dose and next test tracker on main menu ●
- Visual travel-based concept redesign throughout the app ●
- Stickers on therapy time graph to denote more than 30 minutes of therapy dose in a day achieved ●
- Occasional performance-specific motivational messages to pop-up on feedback graphs ●

Theme 4 – Personal Trajectory

- Viewing test results and therapy graphs is optional and decided by the user ●

Theme 5 – Recognisable and Relatable Content

- The numeric point system will be removed and replaced with visual and audio performance feedback through an animated cartoon character to provide immediate performance ●
- In the Challenge Phase, replace complicated '=' and '≠' icons on action buttons with a tick and cross symbols ●

Theme 6 – Social and Sharing

- Development of a feature to enable users to export a therapy progress 'highlights' image to share via email, WhatsApp or text message ●
- Development of a portal to allow clinicians to securely monitor their patients' progress in the therapy. ●

Theme 7 – Widening Participation

- Registration process redesign with visual support through aphasia-friendly symbols and videos. ●
- Have pause pages in between registration testing to allow for breaks. ●
- Aphasia-friendly videos on how to set up a Google Play account and how to start using iReadMore available online and through the app ●
- Aphasia-friendly FAQs page available online and via the app to support use of the therapy ●
- Development of Apple and Android versions of the app to work across the majority of currently available phones and tablets ●
- Written content to be placed in centre of the screen (for users with visual impairments) ●
- All written information is read aloud using real audio recordings (as opposed to using a text-to-speech function) ●
- Setting to enable only Male voices in the therapy (for those with high frequency hearing loss) ●
- Create a demo version of the app for prospective app users ●
- Encrypted contact portal in the app for users to get technical support from the developers ●
